# Convulsions in the treatment of obsessive–compulsive disorder

**DOI:** 10.4103/0019-5545.55945

**Published:** 2005

**Authors:** Soumya Basu, Subhash Chandra Gupta, S. Haque Nizamie

**Affiliations:** * Ex-Senior Resident, Central Institute of Psychiatry, Kanke, Ranchi 834006, India; ** Ex-Junior Resident, Central Institute of Psychiatry, Kanke, Ranchi 834006, India; *** Professor of Psychiatry and Director, Central Institute of Psychiatry, Kanke, Ranchi 834006, India

**Keywords:** Obsessive–compulsive disorder (OCD), seizure, epilepsy, convulsion, electroconvulsive therapy (ECT)

## Abstract

Obsessive–compulsive disorder (OCD) and epilepsy tend to share a close association. However, the exact relationship between OCD symptoms and epileptic convulsions is not well known. A case of OCD who improved remarkably following drug-induced seizures is described, implicating a role for convulsion as an alternative therapeutic modality in OCD.

## INTRODUCTION

The antagonism between epilepsy and psychosis is well known in the literature.^1^ This clinical observation was the rationale behind the introduction of convulsive therapy in psychiatry.^2^ However, electroconvulsive therapy (ECT), regarded as the gold standard for treating depression, is generally viewed as having limited benefit in obsessive–compulsive disorder (OCD), despite isolated reports of its success in treatment-resistant cases.^3^,^4^ The authors describe a case of a patient with OCD who had remarkable improvement following drug-induced seizures, implicating a role for convulsion as an alternative therapeutic modality in OCD.

## THE CASE

A 36-year-old married woman with a family history of OCD in the mother, father and a younger sister, presented to the OPD of the Central Institute of Psychiatry, Ranchi, with a history of occurrence of two attacks of seizures over a period of one year, and a history of psychiatric illness for the past six years characterized by obsessions of dirt and contami-nation, with compulsive ritualistic washing. The patient was on regular psychiatric care for the past 4–5 years and her treatment consisted of medication and behaviour therapy. She was on Tab. clomipramine 150 mg, Cap. fluoxetine 60 mg and Tab. buspirone 10 mg when she had the first attack of generalized tonic–clonic convulsions. During this period, the patient still had florid obsessive–compulsive symptoms as well as secondary depression. However, after the attack of seizure, her obsessive–compulsive symptoms improved by almost 50% and her depression also improved.

After the attack, the dose of clomipramine was reduced to 100 mg and the other medications continued as before. The patient continued to maintain the same clinical improvement. Four months after the first seizure, the patient had a similar attack of seizure. Within two days of this episode, a remarkable improvement in the OCD and depression was noted and, according to the patient and her husband, there was almost 100% improvement. The patient was next seen by a neurologist who started her on 400 mg of Tab. sodium valproate, she discontinued clomipramine on her own but continued to take Cap. fluoxetine 20 mg and Tab. buspirone 10 mg. A CT scan was done, which did not reveal any abnormality. An EEG done after the first attack and a Q-EEG done after the second attack did not show any abnormality. Two months after the seizure, the obsessive-compulsive and depressive symptoms re-emerged. She was symptomatic when she contacted us, her Yale-Brown Obsessive Compulsive Scale (YBOCS) score was 26, indicating a moderate level of symptoms.^5^ The patient's medication was changed to Tab. sertraline 150 mg and she continued on Tab. sodium valproate 400 mg. When last contacted, she was better but had not reached the level of clinical improvement that she experienced after she had her second convulsion.

## DISCUSSION

There is an inseparable relationship between OCD and epilepsy. Obsessive–compulsive-like personality changes are known to occur in long-standing epilepsy.^6^ Forced thinking mimicking obsessive thoughts is known to occur as an aura of frontal lobe seizures.^7^ There have been reports of clinical improvement of OCD in patients with epilepsy on carbamazepine monotherapy.^8^ There have been contradictory findings of cure for OCD^9^ as well as its causation after epilepsy surgery.^10^ However, even after a thorough literature search including a Medline one with the key words OCD, seizure and ECT, we did not find any report on improvement of OCD after seizures. Hence, to our knowledge, this case is the first one reporting an improvement of OCD with spontaneous occurrence of seizures. Similar improvement in psychotic illness is documented in the literature^1^ as already discussed. A similar observation was made in the 1960s when Landolt described forced normalization as a concept to a worsening of psychiatric symptoms when the EEG is normalized by anti-epileptic therapy.^11^ Forced normalization is known to worsen psychosis and depression^11^ but little is known about its relationship with OCD symptoms. The occurrence of seizures in this case in the background of a normal CT scan and EEG can be explained by the use of the combination of clomipramine, fluoxetine and buspirone, each of which is individually known to reduce the seizure threshold and induce seizures.^12^ However, ideally the patient should have had an MRI scan which is more sensitive in detecting cerebral pathology. It could not be done in this case.

An interesting observation here is that an apparent treatment-resistant case of OCD improved abruptly with the accidental onset of seizures. The improvement was more marked after the second attack and the improvement lasted for almost two months. Such a high level of improvement of a chronic illness is remarkable. The clinical improvement of OCD and depression with spontaneous occurrence of generalized tonic–clonic seizures implicates a curative role for convulsions in treatment-resistant cases of OCD and secondary depressive symptoms. Currently, there are a few reports of improvement of treatment-resistant cases of OCD with ECT in the literature.^3^,^4^ In some instances, the favourable response to ECT was short-lived. Khanna *et al*.^13^ described 9 treatment-refractory patients with OCD (without depression) who underwent ECT, resulting in a more than 20% decline in global OCD ratings. However, all returned to their baseline illness by four months. In this case, a similar short-term benefit was noted after the episode of drug-induced seizures. The improvement occurred simultaneously in both the OCD and the depressive symptoms.

In conclusion, in this case, a major improvement of OCD symptoms with epileptic convulsions was observed. Hence, using convulsions in the form of ECT can be suggested as method of treatment. Such a recommendation has also been made in current protocols of treatment for drug-resistant OCD, especially with secondary depression.^4^,^14^
